# Staged Penetrating Sclerokeratoplasty and Penetrating Keratoplasty for Management of Advanced Acquired Anterior Staphyloma

**Published:** 2011-04

**Authors:** Enrique de la Torre-Gonzalez, Carolina Ponce de León Ascencio

**Affiliations:** Hospital Juarez de México, Mexico’s National Autonomous University, Mexico City, Mexico

**Keywords:** Anterior Staphyloma, Sclerokeratoplasty, Penetrating Keratoplasty, Corneal Ulcer

## Abstract

Herein we describe a staged surgical technique consisting of penetrating sclerokeratoplasty (PSKP) followed by penetrating keratoplasty (PKP) and present its clinical course and complications over two years of follow-up. A 23-year-old man presented with cosmetically unacceptable protrusion of the globe corresponding to the cornea and sclera. PSKP was performed transplanting a full-thickness beveled 13 mm corneoscleral tectonic graft. Hypotony developed subsequently and was successfully managed medically, however corneal graft failure occurred. After 15 months, a 7.5 mm PKP was performed for optical reasons, which subsequently remained clear with a healthy epithelium. In this particular case, cosmetic, tectonic, therapeutic, and optical requirements were met. PSKP is a surgical procedure which entails a high rate of complications but may be the only alternative when the main goal of intervention is restoration of the globe in complicated cases such as our patient.

## INTRODUCTION

A staphyloma is a localized defect in the eye wall with protrusion of uveal tissue due to alterations in scleral thickness and structure. Most staphylomas occur in the posterior pole, usually due to pathological or degenerative myopia.

Anterior staphylomas are less frequent. They generally occur after longstanding mistreated or untreated infections in a previously healthy eye and are commonly associated with fungal corneal ulcers. In other cases, they may follow chronic inflammatory diseases such as necrotizing scleritis. Due to their uncommon occurrence, very few case reports in the literature have discussed their management.^1^ These patients often require more than one surgical procedure in a stepwise manner which may take years to complete. Tectonic, therapeutic, cosmetic, and optic implications must be considered in advanced anterior staphylomas, and all must be kept in mind before undertaking the case.

Penetrating sclerokeratoplasty (PSKP) is a surgical technique that consists of replacing the anterior one-third of the eye wall with a full-thickness corneoscleral graft of variable size, depending on the extent of the staphyloma. This procedure was first described in 1956 and achieves a clear graft in 50% of cases over a 3.5 year period.^2^ It may also prove an acceptable technique for providing tectonic support and stabilizing eyes with severe anterior segment ectasia.^3^ Complications include a high rate of rejection and graft failure, secondary glaucoma, chronic epithelial defects, phthisis bulbi, choroidal hemorrhage, and expulsive hemorrhage, among others.^2^ The results of this technique are poor in terms of maintenance of vision.^4^

## SURGICAL TECHNIQUE

**Figure 1:**
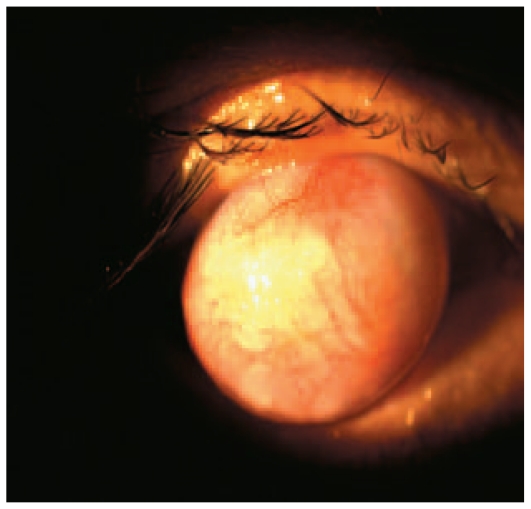
Acquired anterior staphyloma preventing eyelid closure.

A 23-year-old male farmer from Chiapas, Mexico with no history of previous eye surgery was referred with protrusion of his right eye. Two years earlier, the patient had experienced redness in the same eye for which he had received multiple topical and systemic medications with no improvement. The inflammation eventually evolved into the condition he presented with. In addition to poor visual acuity (light perception), the corneoscleral protrusion was cosmetically unpleasant and precluded complete eyelid closure, resulting in chronic foreign body sensation (Fig. 1). Examination of the left eye was unremarkable; this eye had visual acuity of 20/20 with no sign of previous or present inflammation.

**Figure 2:**
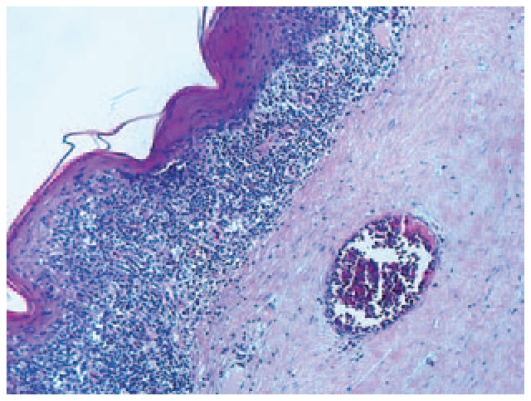
Histopathology of the excised specimen, the corneal epithelium is transformed into epidermis; loss of Bowman’s layer is noted and neovascularization is present in the anterior stroma (Hematoxylin-Eosin, ×40).

The first surgical step was a PSKP with beveled margins. In order to maintain the structure of the recipient angle, both donor and recipient tissues were prepared freehand. The recipient eye underwent a 360° peritomy with relaxing conjunctival incisions. Then, a circumferential 50% deep beveled scleral incision was made 2.5 mm peripheral to the limbus, until the peripheral cornea was reached. Next, a 360° sclerokeratectomy was performed. Figure 2 depicts the histopathology of the excised corneoscleral tissue.

**Figure 3:**
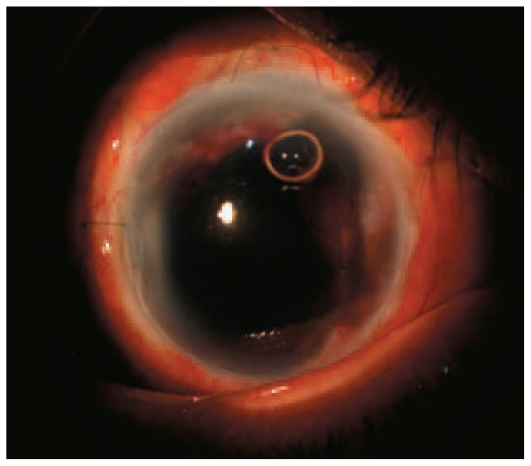
Anterior segment appearance one day after sclerokeratoplasty, note the formed anterior chamber.

The crystalline lens was found to be displaced forward with absent zonular support, leading to spontaneous intracapsular extraction. Anterior dry vitrectomy was performed due to vitreous prolapse following cataract extraction. The donor graft was prepared leaving a 3 mm scleral rim attached to the cornea; the scleral margin was beveled such that a large mushroom-shape pattern was created. The donor graft was sutured to the beveled recipient scleral bed with 10-0 nylon and 7-0 vicryl sutures to promote scleral vascularization. The overlying conjunctiva was sutured with 10-0 nylon sutures. After the procedure, the graft remained clear with a formed anterior chamber and a smooth donor-recipient interface covered with conjunctiva (Fig. 3).

On the second postoperative day, ocular hypertension developed and medical treatment was initiated. After three months (October 2008), hypotony occurred spontaneously and the anti-glaucoma agents were discontinued. B-scan ultrasonography revealed choroidal thickening and treatment with high dose topical and transseptal depot steroids, and 1% atropine was initiated. Six months after the procedure (February 2009), the graft lost transparency (Fig. 5C). Fifteen months after PSKP, the second surgical stage was undertaken while intraocular pressure (IOP) was within normal range without any medications. A 7.5 mm penetrating keratoplasty (PKP) was performed within the previous sclerocorneal graft.

**Figure 4:**
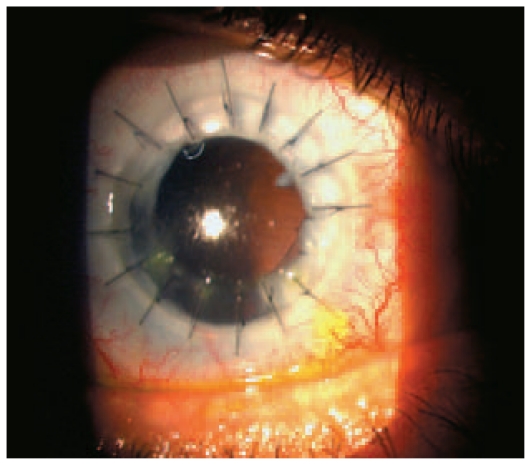
After penetrating keratoplasty the graft remained clear.

**Figure 5:**
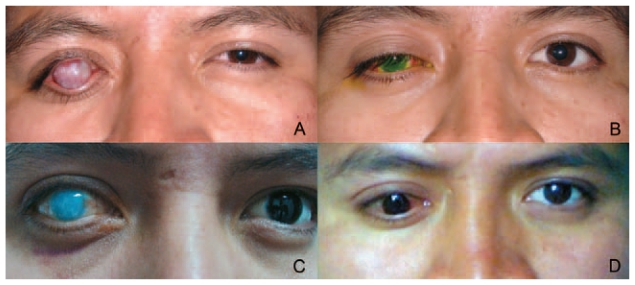
Appearance of the patient, pre- and postoperatively: **(A)** before penetrating sclerokeratoplasty (PSKP), **(B)** post-PSKP, **(C)** after late graft failure, and **(D)** final result with a cosmetic contact lens.

The graft remained clear with a healthy epithelium and normal IOP (Fig 4). At this point, the posterior pole appeared to be normal and visual acuity was counting fingers at 2 m. The patient decided to wear a cosmetic soft contact lens (Fig. 5).

## DISCUSSION

Acquired anterior staphylomas usually follow untreated fungal ulcers in developing countries; nevertheless other microorganisms such as Pseudomonas or Acanthamoeba have also been implicated as the cause.^1^ PSKP is a surgical technique that provides acceptable tectonic outcomes in eyes with severe anterior staphylomas.^3^ Panda et al^6^, in a prospective analysis that included 20 eyes with anterior staphylomas, reported better outcomes following PSKP as compared to PKP during a one-year follow-up period. They also found a lower incidence of ocular hypertension and reinfection with PSKP.^7^ In another study, Jonas et al^8^ reported a number of PSKP complications such as ocular hypotony (as in our patient), glaucoma, loose sutures, persistent epithelial defects, and the need for PKP as a second procedure due to opacification of the first graft.

PKP as a second procedure demonstrates high rates of failure and rejection, and should be considered as a high risk procedure. These concerns may be circumvented by performing a keratoprosthesis^9^ procedure; however, due to need for close and long-term follow-up, and high cost of the latter procedure, we preferred to perform a PKP and achieved good outcomes.

Jacob et al^5^ described a technique using a biosynthetic graft to simulate the anterior segment in a child with diffuse anterior staphyloma. The graft had a biological segment (corneoscleral button) and a synthetic part (aniridic intraocular lens). The authors reported that 6 months after the procedure, the results of the technique were anatomically and cosmetically satisfactory; however, longer follow-up is required to establish long term outcomes.

Acquired anterior staphylomas are rarely seen in developed countries. Low education and poor sanitary conditions in a patient with no access to appropriate health care, may rarely lead to such a condition. The treatment of acquired anterior staphylomas is complex and involves high-risk procedures such as PSKP. Cosmetic, tectonic, therapeutic, and optical considerations must be met and most likely, more than one procedure is needed. PSKP offers excellent tectonic, therapeutic, and cosmetic rehabilitation, albeit with limited optical advantages in the long term. PSKP entails a high rate of complications, but is the only alternative for restoring the globe in advanced cases such as the patient reported herein. Keratoprosthesis may benefit patients if a second surgical procedure is required, but is rarely available and often not reccomended in developing countries.
